# Effect of Yijin-Tang, an Oriental Traditional Formula, on Allergic Responses Using an Ovalbumin-Induced Murine Asthma Model

**DOI:** 10.1155/2021/5585692

**Published:** 2021-05-11

**Authors:** Se-Jin Lee, A. Yeong Lee, Je-Oh Lim, Ji Hye Lee, Tae-Yang Jung, So-Won Pak, Woong-Il Kim, Yoon Soo Seo, Jong-Choon Kim, Je-Won Ko, In-Sik Shin

**Affiliations:** ^1^College of Veterinary Medicine and BK21 FOUR Program, Chonnam National University, 77 Yongbong-ro, Buk-gu, Gwangju 61186, Republic of Korea; ^2^Herbal Medicine Resources Research Center, Korea Institute of Oriental Medicine, 111Geonjae-ro, Naju-si 58245, Jeollanam-do, Republic of Korea; ^3^College of Veterinary Medicine, Chungnam National University, 99 Daehak-ro, Yuseong-gu, Daejeon 34131, Republic of Korea

## Abstract

Yijin-tang is an oriental traditional herb used to treat inflammatory diseases. In the present study, we investigated the protective effects of Yijin-tang water extract (YTE) using an ovalbumin- (OVA-) induced asthma model, focusing on the antioxidant and anti-inflammatory properties of the herb. BALB/c mice were intraperitoneally injected with OVA on days 0 and 14 and then challenged with OVA on days 21, 22, and 23. The animals were orally administered YTE (200 and 400 mg/kg) from days 18 to 23, and this was found to significantly decrease airway hyperresponsiveness and release of inflammatory cells, cytokines, and OVA-specific immunoglobulin E in mice with asthma. In addition, YTE was associated with a marked reduction in airway inflammation and mucus production in lung tissue of mice with asthma. Furthermore, YTE suppressed the expression of matrix metalloproteinase-9 and phosphorylation of ERK in the lungs, which in turn led to a reduction in inducible nitric oxide synthases and an elevation in reduced glutathione and heme oxygenase-1. In conclusion, YTE effectively suppressed allergic responses in mice with asthma and the effect was closely related to antioxidant and anti-inflammatory properties of the herb. Our results indicate that YTE may be a potential agent for the treatment of allergic asthma.

## 1. Introduction

Asthma is a life-threatening disease that affects human health, and its prevalence is increasing annually [1]. It is caused by various allergens, air pollutants, and chemicals, characterized by airway inflammation, airway hyperresponsiveness (AHR), and mucus overproduction [2]. Approximately 300 million people have asthma globally, and its morbidity and mortality are predicted to gradually increase [3]. The mechanisms underlying asthma are known to be very complicated and are associated with various factors such as cytokines, chemokines, and oxidative stress. These factors induce eosinophil-rich inflammation, mucus overproduction, and AHR [1]. Consequently, search has been dedicated to developing asthma treatments based on the various factors that cause asthma [4, 5].

Oxidative stress is closely related to the development and exacerbation of asthma [6]. An imbalance in the oxidant/antioxidant system of an organism triggers the generation of reactive oxygen and nitrogen species (ROS and RNS, respectively), thus worsening asthmatic responses [7]. Therefore, various biological units that decrease oxidative stress are activated, particularly heme oxygenase-1 (HO-1) which acts as an important antioxidant to protect against oxidative stress [8]. Excessive ROS production upregulates HO-1 expression, which eventually decreases inducible nitric oxide synthase (iNOS) and increases antioxidant system mediators including catalase, reduced glutathione (GSH), and superoxide dismutase [9]. This series of events removes ROS and RNS in organisms to reduce oxidative stress, thereby alleviating asthmatic responses [8].

Yijin-tang, an oriental traditional herbal prescription, is mainly used to treat gastrointestinal diseases such as gastritis and gastric ulcers [10]. It is composed of *Citrus unshiu*, *Glycyrrhiza uralensis*, *Pinellia ternate*, *Poria cocos,* and *Zingiber officinale*. According to a previous study, Yijin-tang was postulated to have very strong antioxidant properties [11]. Yijin-tang has been shown to exhibit curative effects against gastric injury induced by ethanol through enhancement of antioxidant status. In addition, the crude herbs that constitute Yijin-tang have strong antioxidant properties [12–15]. Owing to this, we predicted that Yijin-tang would be effective in treating asthma. Moreover, a systematic review and meta-analysis reported that Yijin-tang may be used in the treatment of respiratory diseases [16]. Therefore, we explored the curative effects of Yijin-tang using an ovalbumin- (OVA-) induced asthma model, based on the antioxidant and anti-inflammatory properties of the herb.

## 2. Materials and Methods

### 2.1. Animals

Six-week-old BALB/c female mice were obtained from SAMTAKO (Osan, Republic of Korea). The animals were housed under normal condition (22 ± 2°C, 55 ± 5 % RH, and a 12 h night/day cycle) and received feed and water *ad libitum* for 5 weeks. Study approval was obtained from the Chonnam National University Institutional Animal Care and Use Committee (CNU IACUC-YBR-2020-23, Gwangju, Republic of Korea). Five experimental groups (*n* = 5 per group) were designed: CON (control, PBS administration), OVA (asthma model and PBS administration), DEX (asthma model and dexamethasone administration), and YTE 200 and 400 (asthma model and YTE administration (200 and 400 mg/kg, respectively)).

### 2.2. Plants

Five Yijin-tang herbs, composed of the peel of *Citrus unshiu* Markovich (150 g), root of *Glycyrrhiza uralensis* Fischer (75 g), tuber of *Pinellia ternata* Breitenbach (300 g), sclerotium of *Poria cocos* Wolf (150 g), and rhizome of *Zingiber officinale* Roscoe (3 pieces), were purchased from Kwangmyongdang Pharmaceutical Co. (Ulsan, Republic of Korea) and analyzed for purity by Dr. Goya Choi. The individual herbs were macerated for 30 min and extracted by reflux in 4.8 L of distilled water (D W) at 100°C for 2 h. The solution was filtered through chromatographic paper (46 × 57 cm, Whatman Ltd., Maldstone, England) and freeze-dried to obtain 104.1 g (14.16%, w/w) of Yijin-tang water extract (YTE, voucher no. YJ-1).

### 2.3. Chemicals

Liquiritin (≥98.0%) and narirutin (≥98.0%) were purchased from ChemFaces (Wuhan ChemFaces Biochemical Co., Ltd., Wuhan, China), glycyrrhizic acid (≥95.0%) was obtained from Sigma-Aldrich (Sigma-Aldrich, Seoul, Republic of Korea), and hesperidin was purchased from Wako (Fujifilm Wako Pure Chemical Corporation, Osaka, Japan), high-performance-liquid-chromatography- (HPLC-) grade DW, acetonitrile, and methanol were obtained from Merck (Sigma-Aldrich, Darmstadt, Germany).

### 2.4. HPLC Analysis

Quantitative analysis of compounds containing YTE was performed using an HPLC system (Waters Corporation, Milford, MA, USA) coupled to a 2998 PDA detector, separation module (Waters e2695), micro slitter (IDEX Health and Science LLC., Oak Harbor, WA, USA), and Acquity QDa^TM^ detector. Analytical data were processed using Empower 3 software (Waters Corporation). YTE (121.25 mg) dissolved in DW (10 mL) was injected using a 0.2 *μ*m syringe filter, and constituents of YTE were separated using an Xselect HSS T3 5 *μ*m analytical column (250 mm × 4.6 i.d) (Waters Corporation) using a mobile phase flowrate of 0.8 mL/min. The mobile phase consisted of 0.05% formic acid in DW (A), methanol (B), and acetonitrile (C), and the linear gradient program was as follows: 100% A–92% A (7% B and 1% C) for 0–3 min; 92% A (7% B and 1% C)–70% A (25% B and 5% C) for 3–16 min; 70% A (25% B and 5% C)–60% A (32% B and 8% C) for 16–30 min; 60% A (32% B and 8% C) isocratic for 30–35 min; 60%A (32% B and 8% C)–55% A (36% B and 9% C) for 35–45 min; and 55% A (36% B and 9% C)–0% A (70% B and 30% C) for 55–62 min. The single quadruple mass detector (QDa detector) setting was used with a probe temperature of 600°C, an ESI capillary voltage of 0.8 kV, con voltage of 15 V, 120°C, and a split ratio of 10 : 1. The temperatures of the auto-sampler and analytical column were at 10°C and ambient, respectively. The sample injection volume was 10 *μ*L, and wavelengths were scanned from at 195–400 nm. Sample peaks were detected at 254 nm (for glycyrrhizic acid) and 280 nm (for liquiritin, narirutin, and hesperidin). The wavelengths and molecular weights of compounds found in YTE were compared against ultraviolet (UV) and mass spectrometry (MS) spectra data of respective analytical standards.

### 2.5. Experimental Procedure

The OVA-induced asthma model was established according to a previous study [1]. The animals were intraperitoneally injected with OVA (20 *μ*g, Sigma-Aldrich, St. Louis, MO, USA) containing aluminum hydroxide (2 mg, Sigma-Aldrich) on days 0 and 14. Subsequently, animals were administered 1% OVA for 1 h via inhalation from days 21 to 23. Dexamethasone (1 mg/kg) and YTE (200 and 400 mg/kg) were orally administered from days 18 to 23. Airway hyperresponsiveness (AHR) was estimated on day 24, and mice were sacrificed on day 25. Blood was collected from the caudal vena cava of each animal and then centrifuged at 200 ×g for 20 min to separate out the serum.

### 2.6. Measurement of AHR

On day 24, we evaluated AHR by whole-body plethysmography (OCP3000, Allmedicus, Seoul, Republic of Korea). AHR was measured following aerosolization of PBS and increased concentrations of methylcholine (10, 20, and 40 mg/mL, Sigma-Aldrich) in PBS for 3 min. The data are shown as the dimensionless parameter enhanced pause (Penh).

### 2.7. Analysis of BALF and Serum

Bronchoalveolar lavage fluid (BALF) sampling was performed as previously described [1]. Briefly, an endotracheal tube was instillated into the trachea of each animal after tracheostomy. PBS (0.7 mL) was administered into the left lung, and samples were transferred to the tube; this was repeated once more (to obtain 1.4 mL of BALF). The BALF samples were centrifuged at 200 ×g for 10 min, and the supernatant (1.2 mL) was stored at −20°C for future use in evaluating cytokines. The number of total cells in BALF was evaluated using a cell counter (Countess 3 Automated Cell Counter, Thermo Fisher Scientific, Waltham, MA, USA). To perform visible inflammatory count, BALF pellets were dissolved in PBS (200 *μ*L), inflammatory cells were attached using centrifugation (Hanil, Wonju, Republic of Korea), and the slides were stained with Diff-Quik reagent (Sysmex, Kobe, Japan). The number of differential inflammatory cells in BALF pellets was obtained by applying the ratio of visible inflammatory cells to the number of total cells. BALF supernatant was evaluated for levels of interleukin- (IL-) 4, -5, and -13 using commercial ELISA kits (R&D System, Minneapolis, MN, USA). In serum, we evaluated the levels of OVA-specific IgE by ELISA according to previous studies [1, 4].

### 2.8. Histopathology

The left lung of each animal was fixed in neutralized buffered formalin, embedded, and sections (4 *μ*m) were prepared. Sections were stained using hematoxylin and eosin (Sigma-Aldrich) and periodic acid-Schiff (IMEB Inc., San Marcos, CA, USA). Quantitative analysis of inflammatory responses and mucus production was conducted using an image analyzer (IMT i-Solution Inc., Vancouver, BC, Canada). To evaluate the expressions of HO-1 and matrix-metalloproteinase-9 (MMP-9) in the lungs, we performed immunohistochemistry (Vector Laboratories, Burlingame, CA, USA) according to previous studies [4, 17]. Anti-mouse HO-1 antibody (Abcam, Cambridge, UK) and anti-mouse MMP-9 (Abcam) were used as the primary antibodies.

### 2.9. Western Blotting

To investigate protein expression related to inflammation and oxidative stress, we performed western blotting according to a previous study [17]. The antibodies of phosphorylated-ERK and HO-1 were obtained from Abcam Co., and MMP-9, iNOS, GSH, and *β*-actin were purchased from Cell Signaling (Beverly, MA, USA). The quantitative analysis of each protein band was measured using ChemiDoc (Bio-Rad Laboratories, Hercules, CA, USA).

### 2.10. Statistical Analysis

All data are presented as mean ± standard deviation. Statistical evaluation was performed using analysis of variance (ANOVA) followed by Dunnett`s post hoc adjustments. *p* values <0.05 and <0.01 were considered to be statistically significant.

## 3. Results

### 3.1. HPLC Analysis of YTE

**Figure 1:**
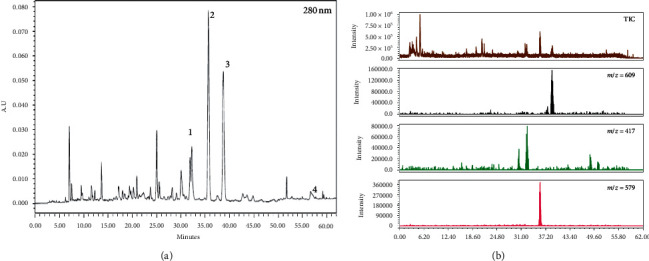
Chromatogram of YTE at 280 nm (a) and total ion chromatogram and selected ion chromatogram (b).

The HPLC chromatogram of YTE is depicted in Figure 1. Four compounds, liquiritin (1), narirutin (2), hesperidin (3), and glycyrrhizic acid (4), were detected at approximately 32.3, 35.7, 38.7, and 57.3 min (Figure 1(a)), respectively. Quantitation of liquiritin, narirutin, hesperidin, and glycyrrhizic acid yielded 1.56 ± 0.00842, 4.19 ± 0.0125, 3.58 ± 0.0304 *μ*g/mg, and trace, respectively. UV absorption spectra of these compounds showed *λ*_max_ values of 275.9, 283.1, 284.2, and 249.9 nm, respectively. Figure 1(b) shows the total ion chromatogram and extracted ion chromatogram of the molecules. MS values (*m/z*) in the negative mode of the mass spectrum were confirmed as follows: liquiritin [M-H]^−^ = 417.10, narirutin [M-H]^−^ = 579.14, hesperidin [M-H]^−^ = 609.19, and glycyrrhizic acid [M-H]^−^ = 821.22.

### 3.2. Effect of YTE on AHR in Mice with Asthma

**Figure 2:**
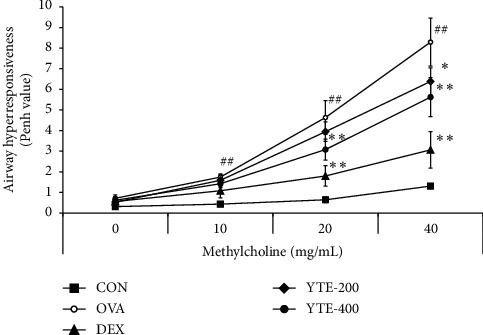
Effect of YTE on AHR in an OVA-induced asthma mouse model. AHR was regarded as a crucial clinical sign exhibiting respiratory contraction in asthma and evaluated using whole-body plethysmograph. CON, PBS administration; OVA, asthma model and PBS administration; DEX, asthma model and dexamethasone administration; YTE-200 and -400, OVA asthma model and YTE administration. ^##^*p* < 0.01 vs CON; ^*∗*^^*∗∗*^*p* < 0.05 and 0.01 vs. OVA, respectively.

The OVA group exhibited a significant elevation in AHR compared with the CON group (Figure 2). In contrast, the DEX group showed a markedly decreased AHR in comparison to the OVA group. Similarly, YTE groups also showed a notable reduction in AHR in comparison to the OVA group; the decrease was more evident in the YTE-400 group.

### 3.3. Effect of YTE on Accumulation of Inflammatory Cells in BALF of Mice with Asthma

**Figure 3:**
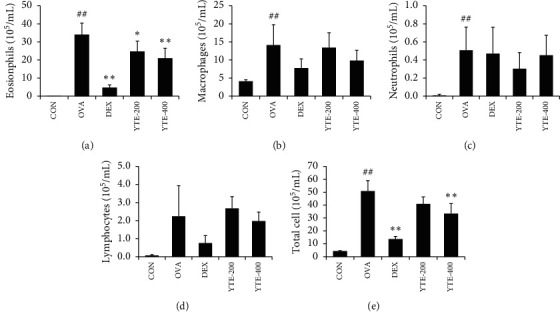
Effect of YTE on accumulation of inflammatory cells in BALF of mice with asthma. Inflammatory cell count was markedly increased in the development of asthma. (a) eosinophils, (b) macrophages, (c) neutrophils, (d) lymphocytes, and (e) total cells. CON, PBS administration; OVA, asthma model and PBS administration; DEX: asthma model and dexamethasone administration; YTE-200 and -400, asthma model and YTE administration. ^##^*p* < 0.01 vs. CON; ^*∗*^^*∗∗*^*p* < 0.05 and 0.01 vs. OVA, respectively.

The OVA group showed a marked elevation in inflammatory cell count in comparison to the CON group (Figure 3). Particularly, the elevation in BALF eosinophil count was detected in the OVA group. In contrast, the DEX group exhibited a noticeable reduction in BALF inflammatory cell count in comparison to the OVA group. Similar to the results of the DEX group, YTE groups showed a notable reduction in inflammatory cell counts in comparison to the OVA group, however, the decline being more evident in the YTE-400 group.

### 3.4. Effect of YTE on Cytokine Levels and OVA-Specific IgE in Mice with Asthma

**Figure 4:**
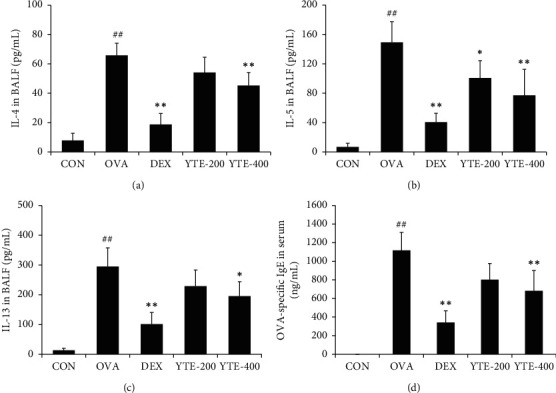
Effect of YTE on the levels of cytokines and OVA-specific IgE in mice with asthma. The cytokines were associated with the allergic responses such as eosinophilia, AHR, and IgE production. (a) IL-4, (b) IL-5, (c) IL-13, and (d) OVA-specific IgE. CON, PBS administration; OVA, asthma model and PBS administration; DEX, asthma model and dexamethasone administration; YTE-200 and -400, asthma model and YTE administration. ^##^*p* < 0.01 vs. CON; ^*∗*^^*∗∗*^*p* < 0.05 and 0.01 vs. OVA, respectively.

The OVA group showed a notable elevation in BALF IL-4 level in comparison to the CON group (Figure 4(a)). In contrast, the DEX group significantly decreased IL-4 level as compared to the OVA group. In addition, the YTE groups exhibited a significant reduction in BALF IL-4 levels in comparison to the OVA group in a dose-dependent manner. Consistent with the results of IL-4, the levels of IL-5 and -13 markedly decreased in the YTE groups compared to those observed in the OVA group (Figures 4(b) and 4(c), respectively). The levels of OVA-specific IgE were markedly elevated in the OVA group in comparison with those seen in the CON group (Figure 4(d)). Conversely, the YTE groups exhibited substantially reduced OVA-specific IgE level compared with the OVA group. The YTE-400 group showed a notable decline in OVA-specific IgE levels in comparison to the OVA group.

### 3.5. Effect of YTE on Inflammation and Mucus Production in Mice with Asthma

**Figure 5:**
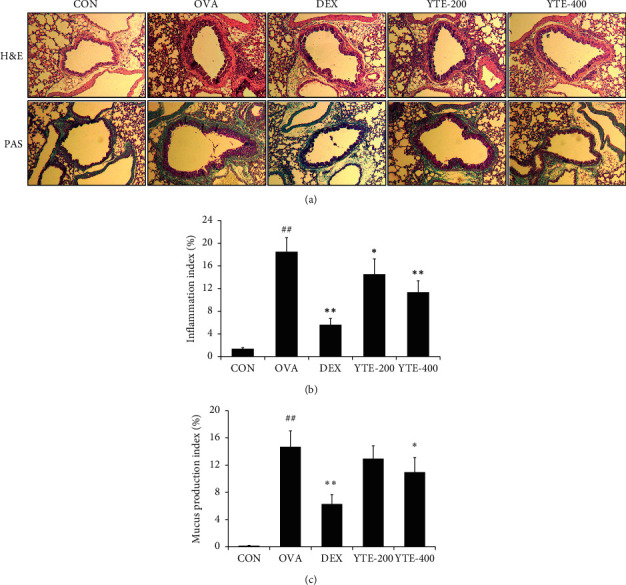
Effect of YTE on airway inflammation and mucus production in mice with asthma. Allergic asthma was featured as airway inflammation and mucus production in respiratory tract. (a) Histological figure of lung tissue, (b) inflammation Index, and (c) mucus production index. CON, PBS administration; OVA, asthma model and PBS administration; DEX, asthma model and dexamethasone administration; YTE-200 and -400, asthma model and YTE administration. ^##^*p* < 0.01 vs. CON; ^*∗*^^*∗∗*^*p* < 0.05 and 0.01 vs. OVA, respectively.

The OVA group exhibited a marked accumulation of inflammatory cells in lung tissue in comparison to the CON group (Figures 5(a) and 5(b)). However, the DEX group showed a notable reduction in airway inflammation in comparison to the OVA group. Consistent with results of the DEX group, YTE groups showed significant decreases in accumulation of inflammatory cell in lung tissue in comparison to the OVA group in a dose-dependent manner. Furthermore, the OVA group showed a notable elevation in mucus production compared with the CON group (Figures 5(a) and 5(c)). On the contrary, the DEX group exhibited significantly decreased mucus production compared with the OVA group. The YTE groups showed decreased mucus production in comparison to the OVA group. In particular, the YTE-400 group showed a significant reduction in mucus production in comparison to the OVA group.

### 3.6. Effect of YTE on Antioxidant Signaling in Mice with Asthma

**Figure 6:**
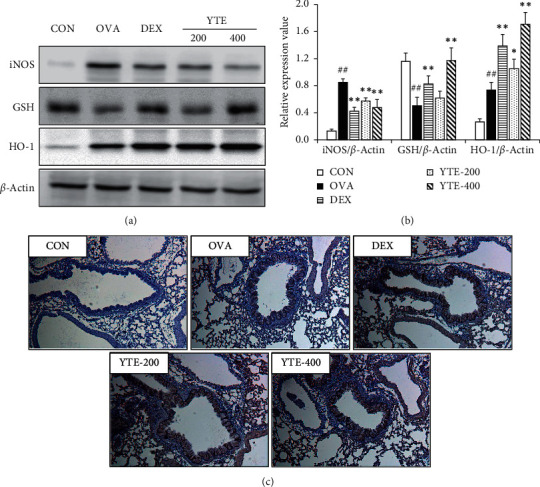
Effect of YTE on antioxidative signaling in mice with asthma. HO-1 acts as an antioxidant enzyme and decreases asthmatic responses. (a) Protein expression on gel, (b) relative expression value, and (c) representative figure for lung tissue following immunohistochemistry (IHC) of HO-1. CON, PBS administration; OVA, asthma model and PBS administration; DEX, asthma model and dexamethasone administration; YTE-200 and -400, asthma model and YTE administration. ^##^*p* < 0.01 vs. CON; ^*∗*^^*∗∗*^*p* < 0.05 and 0.01 vs. OVA, respectively.

The OVA group exhibited a marked increase in the expression of iNOS expression in comparison to the CON group (Figures 6(a) and 6(b)). However, the YTE groups showed a notable reduction in iNOS expression in comparison to the OVA group. The expression levels of GSH remarkably decreased in the OVA group in comparison to those observed in the CON group, whereas a significant increase was observed in YTE groups compared with the OVA group. In addition, the OVA group exhibited an elevation in the expression of HO-1 in comparison to the CON group (Figures 6(a) and 6(c)). Moreover, the expression levels of HO-1 were significantly increased in YTE groups compared to the OVA group.

### 3.7. Effect of YTE on Inflammatory Signaling in Mice with Asthma

**Figure 7:**
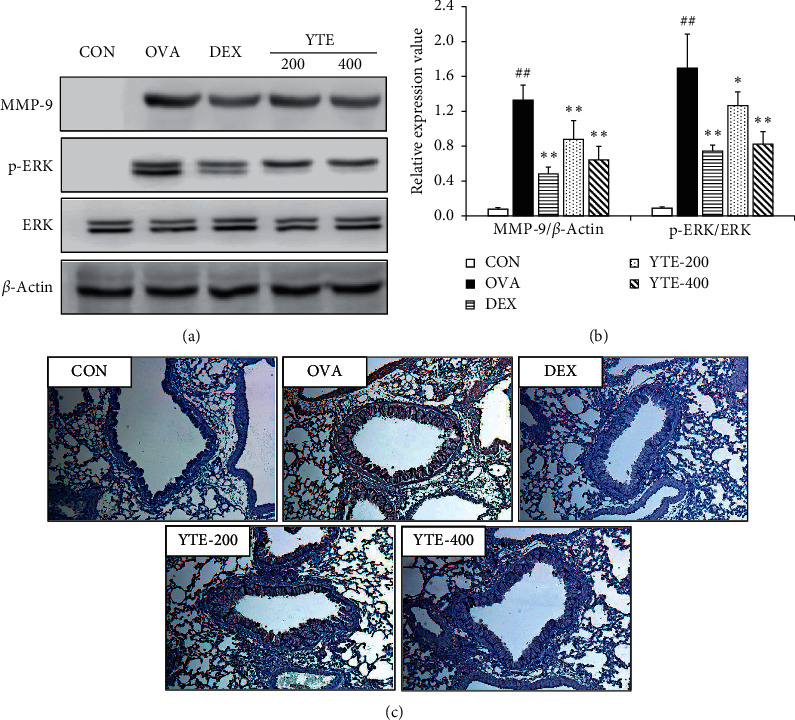
Effect of YTE on the inflammatory signaling in mice with asthma. MMP-9 and ERK were associated with the development of inflammatory response in asthma. (a) Protein expression on gel, (b) relative expression value, and (c) representative figure for lung tissue following IHC of MMP-9. CON, PBS administration; OVA, asthma model and PBS administration; DEX, asthma model and dexamethasone administration; YTE-200 and -400, asthma model and YTE administration. ^##^*p* < 0.01 vs. CON; ^*∗*^^*∗∗*^*p* < 0.05 and 0.01 vs. OVA, respectively.

The OVA group exhibited a notable elevation in the expression levels of MMP-9 compared to the CON group (Figures 7(a)–7(c)). In contrast, the YTE groups showed a notable reduction expression levels of in MMP-9 in comparison to the OVA group in a dose-dependent manner. Furthermore, the OVA group markedly increased the phosphorylation of ERK in comparison to the CON group. However, the YTE groups, in a dose-dependent manner, significantly declined the phosphorylation of ERK in comparison to the OVA group.

## 4. Discussion

Asthma is a chronic inflammatory disorder with a high global incidence and is currently considered a serious life-threatening condition in those with respiratory diseases caused by fine dust, bacteria, and viruses [2, 18, 19]. In the present study, we explored the therapeutic effects of YTE on asthmatic responses using an OVA-induced asthma model. The administration of YTE significantly suppressed the elevation of AHR, cytokines, eosinophils, and OVA-specific IgE in mice with asthma, in addition to reducing airway inflammation and mucus production in the lungs tissue. Furthermore, the administration of YTE significantly decreased the phosphorylation of ERK and the expression of MMP-9 in mice, resulting in an elevation in the expression levels of HO-1 and GSH and a reduction in iNOS.

Eosinophilia is an important feature in the advancement of asthma [20]. Eosinophils are regarded as crucial biomarkers of allergic response and are closely related to inflammatory cytokines, including IL-4, -5, and -13, during the progression of asthma [21]. Cytokines not only trigger not the release of eosinophils into damaged lesions but also aid in the maturation and activation of eosinophils [20]. The activation of eosinophils triggers the releases of various stimulating factors such as ROS, RNS, cytokines, chemokines, and growth factors in granules, eventually triggering AHR, airway inflammation, and mucus overproduction [22]. In this study, administration of YTE reduced the production of inflammatory cytokines, which subsequently decreased the infiltration of inflammatory cells into lung tissue and the production of OVA-specific IgE. Through this cascade, YTE caused a reduction in AHR and inflammatory responses in lung tissue. These results suggest that YTE had a therapeutic effect on the allergic response that occurred in OVA-sensitized mice.

Airway inflammation is caused by various proinflammatory factors. In particular, MMP-9 is a critical mediator in the release of inflammatory cells into inflammatory lesions [23]. MMP-9 promotes airway remodeling by facilitating the degradation of various substrates that make up normal alveolar tissue and induces the release of inflammatory cells into damaged lesions, which ultimately aggravates airway inflammation [24]. The expression of MMP-9 is associated with the phosphorylation of ERK. ERK is a mitogen-activated protein kinase MAPK and is regarded as a critical factor in inflammatory responses; its phosphorylation markedly increases the expression levels of MMP-9 during the progression of asthma [25]. According to a previous study [26], an increase in RK phosphorylation caused an upregulation of MMP-9 expression, which elevated the levels of various inflammatory mediators. In this study, administration of YTE decreased the expression levels of MMP-9 in mice with asthma, as well as reducing ERK phosphorylation. These results suggest that the observed anti-inflammatory properties of YTE could be due to the downregulation of ERK phosphorylation and expression of MMP-9.

Excessive production of ROS is a crucial factor in the progression of asthma [27]. Airway inflammation was found to cause the elevation of ROS production in airway epithelia, eosinophils, macrophages, and neutrophils, which were related to the damage of various biological molecules [28]. In addition, cytokines were found to be associated with the production of ROS [29]. Furthermore, accumulation of ROS was shown to induce AHR by decreasing the function of *β*-adrenergic receptors, releasing histamine from mast cells, and increasing mucus secretion from goblet cells [27]. Moreover, ROS was reported to cause airway epithelial barrier damage, increasing the permeability of inflammatory cells and mediators, resulting in aggravation of airway inflammation [27, 30]. On the contrary, numerous antioxidant biological molecules or systems against oxidative stress exist. HO-1 is part of an antioxidant system that reduces oxidative stress by elevating antioxidant materials such as GSH, resulting in the attenuation of inflammatory responses [31, 32]. Therefore, upregulation of HO-1 expression causes an increase in antioxidant status in organisms, thereby reducing oxidative stress and eventually alleviating airway inflammation, a major feature of asthma, via reducing the production of inflammatory mediators [33, 34]. In this study, administration of YTE resulted in a significant elevation in expression levels of HO-1 in asthma-induced mice, which was accompanied by an increase and decrease in expression levels of GSH and iNOS, respectively. These results showed that YTE reduced asthmatic responses in mice through the upregulation of HO-1 expression.

Antiasthmatic effects of YTE observed in this study were supported by various previous studies [11, 12, 16]. The crude herbs that make up YTE have been shown to have anti-inflammatory and antioxidant properties in various research studies [12–15]. Furthermore, we characterized the active components in YTE using HPLC. Liquiritin, narirutin, hesperidin, and glycyrrhizic acid were detected as the active constituents of YTE. These compounds have been reported to possess anti-inflammatory and antioxidant properties in various studies in the literature [35–38]. Therefore, it is thought that the antiasthmatic effects observed from the use of YTE are closely associated with the aforementioned active moieties.

This study showed that YTE possesses effects that support a favorable prognosis of asthma. However, it is difficult to single out which constituents of YTE exhibit effects that manage the symptoms of asthma. Therefore, it is vital to conduct more experiments, including *in vitro* and *in vivo* studies for each YTE constituent, to determine the active compound(s) with antiasthmatic effect. Moreover, the anti-inflammatory mechanism of YTE identified in this study needs to be clearly demonstrated through genetically engineered mice or cells.

## 5. Conclusions

Overall, YTE decreased asthmatic responses including AHR and release of inflammatory cells, inflammatory cytokines, and OVA-specific IgE levels in asthma-induced mice. These properties of YTE were related to the inhibition of ERK phosphorylation and upregulation of HO-1 expression. Therefore, the study findings suggest that YTE has the ability to be used as a potential antiasthmatic agent.

## Data Availability

The data used to support the findings of this study are available from the corresponding author upon request.
